# Early hemodynamic performance of the Trifecta™ surgical bioprosthesis aortic valve in Indian patient population: 12 month outcomes of the EVEREST post-market study

**DOI:** 10.1186/s13019-018-0783-9

**Published:** 2018-09-25

**Authors:** Gopichand Mannam, Yugal Mishra, Rajan Modi, Alla Gopala Krishna Gokhale, Rajan Sethuratnam, Kaushal Pandey, Rajneesh Malhotra, Sumit Anand, Anushreeta Borah, Sushan Mukhopadhyay, Dhiren Shah, Tek Singh Mahant

**Affiliations:** 1 Department of cardiac surgery, Star Hospital Banjara Hills, 8-2-596/5, Road No.10, Banjara Hills, Hyderabad, Telangana 500034 India; 2 Department of cardiac surgery, Escorts Heart Institute and Research Centre, New Delhi, India; 3 Department of cardiac surgery, SAL Hospital, Ahmedabad, India; 4 Department of cardiac surgery, Yashoda Hospital, Secunderabad, India; 5 Department of cardiac surgery, The Madras Medical Mission, Chennai, India; 6 Department of cardiac surgery, P. D. Hinduja National Hospital & Medical Research Center, Mumbai, India; 7 Department of cardiac surgery, Max Super Speciality Hospital, New Delhi, India; 8 Abbott Pvt. Ltd, New Delhi, India; 9 Department of cardiac surgery, Apollo Gleneagles Hospitals , Kolkata, India; 10 Department of cardiac surgery, Care Institute of Medical Sciences, Ahmedabad, India; 11 Department of cardiac surgery, Fortis Hospital, Mohali, India

**Keywords:** Aortic valve replacement, Stented bioprosthesis, Valvular disease, Structural valve dysfunction

## Abstract

**Background:**

Indian patients undergoing surgical aortic valve replacement (SAVR) differ from western populations with respect to aortic annulus size and valve disease morphology. The purpose of this post-market, non-randomized observational study was to evaluate the early hemodynamic performance of the Trifecta™ bioprosthesis (Abbott, previously St. Jude Medical, Minneapolis, US) in an Indian patient population.

**Methods:**

From January 2014 to September 2015, 100 patients (mean age 64.4 ± 7.1 years, 62% male) undergoing SAVR for valve disease (68% stenosis, 7% insufficiency, 25% mixed pathology) were enrolled across 10 centers in India. Patients implanted with a 19–27 mm Trifecta™ valve were eligible to participate and were prospectively followed for 12-months post-implantation. Echocardiographic hemodynamic performance was evaluated at pre-implant, pre-discharge and at 12-months by an independent core laboratory. Adverse events were adjudicated by the study sponsor. Functional status at 12-months was assessed according to NYHA classification. Continuous data was summarized using descriptive statistics (mean &standard deviation,) and categorical data was summarized using frequencies and percentages.

**Result:**

Ninety patients (mean age 64.5, 62.2% male) completed the 12-month follow up. Significant improvements in hemodynamic valve performance were reported in 81 patients with available echocardiographic data at 12 months. Compared to baseline at 12-month follow up visit, mean effective orifice area increased from 0.75cm^2^ to 1.61cm^2^ (*p* < 0.0001), mean pressure gradient reduced to 10.42 mmHg from 51.47 mmHg (*p* < 0.0001), cardiac output increased from 4.46 l/min to 4.85 l/min (*P* 0.9254). Compared to baseline, functional status improved by ≥1 NYHA class in 75% of patients at 12 months (95% Clopper-Pearson (Exact) confidence limit [64.6%, 83.6%]). No instances of early mortality (< 30 days from index procedure) or structural valve dysfunction were reported.

**Conclusion:**

In an Indian patient population, implantation of the Trifecta™ bioprosthesis is shown to be safe and associated with favorable early hemodynamic performance and improved functional status at 12 months.

**Trial registration:**

The clinical study has been registered under Clinical Trial Registry-India (http://www.ctri.nic.in) and registration number is CTRI/2014/02/004434 registered on 25 February 2014 retrospectively registered.

## Background

Each year, approximately 150,000 patients undergo cardiac surgeries and 30% is the valve surgeries including both aortic and mitral valve replacement in India [1]. Compared to western populations, Indian patients indicated for SAVR tend to be younger, have a higher incidence of valve disease due to rheumatic fever and require implantation of smaller valve sizes (predominately 19 mm and 21 mm valves) [2–4]. Due to the need for implantation of smaller bioprostheses, the risk for patient-prosthetic mismatch (PPM), defined as an effective orifice area (EOA) that is too small in relation to a patient’s cardiac output requirements, is high in Indian patients. PPM leads to poor hemodynamic valve performance (namely elevated transvalvular pressure gradients) despite a fully functioning prosthesis and is associated with poor clinical outcomes including late survival, freedom from heart failure, and LV mass regression [5]. Thus making selection of an appropriate bioprosthesis that maximizes EOA critical in this patient population.

The Trifecta™ bioprosthesis (Abbott, previously St. Jude Medical, Minneapolis, US) is a tri-leaflet, stented, bovine pericardial valve designed for supra-annular placement in the aortic position. The Trifecta^TM^ valve has been commercially available in India since 2012 and incorporates several novel design features to maximize valve hemodynamics while minimizing leaflet stresses [6]. Specifically, the valve features a true supra-annular polyester sewing cuff with a silicone insert that is designed to conform to the shape of the native annulus and externally-mounted bovine pericardial valve leaflets that wrap around the fatigue resistance, titanium stent frame to maximize EOA and improve hemodynamic performance. To reduce the risk of leaflet abrasion and structural valve dysfunction, the stent frame, excluding the sewing cuff, is covered with porcine pericardial tissue to allow only tissue-to-tissue contact during valve function. Linx™ AC anti-calcification technology also reduces calcification of the tissue valve.

The superior hemodynamic performance of Trifecta™ valve has been previously demonstrated in western patients with significant benefits in hemodynamic performance, EOAs and mean transvalvular pressure gradients reported in patients with different annulus sizes [7–12].

Due to the limited data available on Indian patients undergoing SAVR in rheumatic heart disease and with most of the patients having significantly smaller native aortic annuli, the EVEREST study (A clinical EValuation of hEmodynamic peRformancE of Trifecta™ valve in Indian SubjecTs), was conducted with a purpose to evaluate the early (12 months) hemodynamic performance of the Trifecta™ valve by echocardiography in Indian patients for treatment of aortic valve disease. Assessment of the early safety of the valve and changes in NYHA functional classification was also performed.

## Methods

### Patients

Between January 2014 to September 2015, 100 patients who had undergone SAVR with a Trifecta™ valve were recruited from 10 investigational centers in India to participate in the prospective, single-arm, post-market observational EVEREST study (CTRI/2014/02/004434). Eligibility criteria included implantation of a Trifecta™ valve within 7 days and analyzable echocardiographic data at baseline. Patients who had been previously implanted with a prosthetic valve(s) at a site other than the aortic valve, on renal dialysis, pregnant, active endocarditis or pre-existing cardiovascular abnormalities (aortic dissection or ventricular aneurysm) were excluded.

Prior to patient enrollment, appropriate institutional review board approval for protocol, patient information sheets and consent forms were obtained at each center. The study was performed in accordance with the Declaration of Helsinki and with laws and regulations of the country. A written informed consent was obtained from each patient prior to enrollment. Details on the 10 investigational centers are provided in Appendix.

### Surgical technique

The study protocol permitted operative surgeon’s to use any surgical technique at their discretion for implantation of the Trifecta^TM^ valve. Data on valve size, surgical approach, suture technique, cardiopulmonary bypass time and aortic cross clamp time were collected. Post- operative protocol was as per Institute’s standard protocol with anticoagulant and other standard medications.

### Data collection

Preoperative and procedural data was retrospectively collected from the implant centers. Perioperative data collection included patient demographics and medical history, NYHA functional class, disease valve etiology, valve dysfunction (insufficiency/incompetent/regurgitated, stenosis & mixed), echocardiographic exam and pathology. Procedural data collection included implanted bioprosthetic valve size, suture technique, cardiopulmonary bypass time and aortic cross clamp time. All follow-up data was prospectively collected as part of routine clinical practice. At pre-discharge, 6 month and 12-month follow-up visits, subjects underwent a physical examination, echocardiographic exam (pre-discharge and 12 months only), assessment of NYHA functional class and review of relevant cardiac medication.

### Echocardiography

Transthoracic echocardiographic examinations were performed on-site at baseline, pre-discharge (average 6 days of index procedure) and at 12 months by an experienced Echocardiographer. All exams were reviewed by an independent core laboratory by Department of Non-Invasive Cardiology at Fortis Escorts Heart Institute, New Delhi and evaluated for the following parameters: left ventricular function, mean transvalvular pressure gradient, mean pressure gradient, effective orifice area (EOA), cardiac output, cardiac index, and performance index (EOA/Pre-implant Interval Orifice area) [13, 14]. EOA was calculated using the continuity equation [13–15] and indexed to body surface area (indexed EOA). The incidence and severity of aortic insufficiency (paravalvular, valvular or indeterminate with clinical (visual) estimation of regurgitation as trivial, mild, moderate or severe) was assessed according to standardized VARC criteria.

### Endpoints

The study’s primary endpoint was valve hemodynamic performance (as measured by echocardiography) at 12 months. Secondary endpoints included change in NYHA functional class from baseline to 12 months (defined as no change, improved, or worsened), incidence of structural valve deterioration (calcification, leaflet tear and/or perforation) and cardiovascular-related adverse events at 12 months. The study sponsor (Abbott) was responsible for adjudication of adverse events and determining their seriousness and relationship to the study device and or study procedure.

### Statistical analysis

Continuous data was summarized using descriptive statistics (mean &standard deviation) and categorical data was summarized using frequencies and percentages. All echocardiographic parameters were stratified according to the nominal size of the implanted Trifecta™ valve. Comparisons of change between 12-months and baseline in echocardiographic evaluation of valve performance was analysed using Student’s t test (if Normal distribution assumption is met) or Wilcoxon signed-rank test (if Normal distribution assumption is not met). The 95% exact confidence interval is provided to the percentage of the patient with improved NYHA functional between 12-months and baseline. Statistical analyses were performed using SAS™ software v9.4.

## Results

### Patient population and operative data

Baseline clinical and demographic data of the 100 patients enrolled in the study is presented in Table 1. Briefly, patients were predominately male (62%) elderly (mean age 64.38 ± 7.05 years) and had multiple comorbidities including hypertension (51%), coronary artery disease (38%) and diabetes (38%). The main indication for SAVR was valve disease due to degenerative calcification (54%); with only 14% due to rheumatic heart disease. Preoperative NYHA functional class was distributed as 5% Class I, 52.04% Class II, 39.8% Class III and 3% Class IV. Sinus rhythm was observed in 97 patients while 3 patients had a pre-existing arrhythmia.

**Table 1:** Baseline demographics, medical history and clinical characteristics of patients (*n* = 100)

	All (*n* = 100)
Age (years)	64.38 ± 7.05
Male n/N, (%)	62/100 (62%)
Body Mass Index (kg/m^2^)	26.5 ± 4.72
Body Surface Area (m^2^)	1.73 ± 0.18
NYHA functional class	
I	5/98(5.1%)
II	51/98(52.04%)
III	39/98(39.8%)
IV	3/98(3.06%)
Sinus rhythm	97/100 (97%)
Comorbidities	
Hypertension	51/100 (51%)
Coronary Artery Disease	38/100 (38%)
Non aortic Valve Disease	29/100 (29%)
Diabetes mellitus	38/100 (38%)
Hyperlipidemia	3/100 (3%)
Renal Insufficiency	2/100 (2%)
Congestive Heart Failure	2/100 (2%)
Stroke	1/100 (1%)
Transient Ischemic Attack	2/100 (2%)
Myocardial Infarction	1/100 (1%)
Previous coronary artery intervention	4/100 (4%)
Previous CABG	7/100 (7%)
Permanent pacemaker	1/100 (1%)
Aortic Valve Pathology	
Degenerative calcification	54/100 (54%)
Bicuspid	42/100 (42%)
Rheumatic	14/100 (14%)
Endocarditis	1/100 (1%)
Structural deterioration	1/100 (1%)
Other	4/100 (4%)
Aortic Valve Lesion	
Insufficiency	7/100 (7%)
Stenosis	68/100 (68%)
Mixed	25/100 (25%)

*n* Total number of patients, *kg/m*^*2*^ Kilogram/meter^2^

**Fig. 1:**
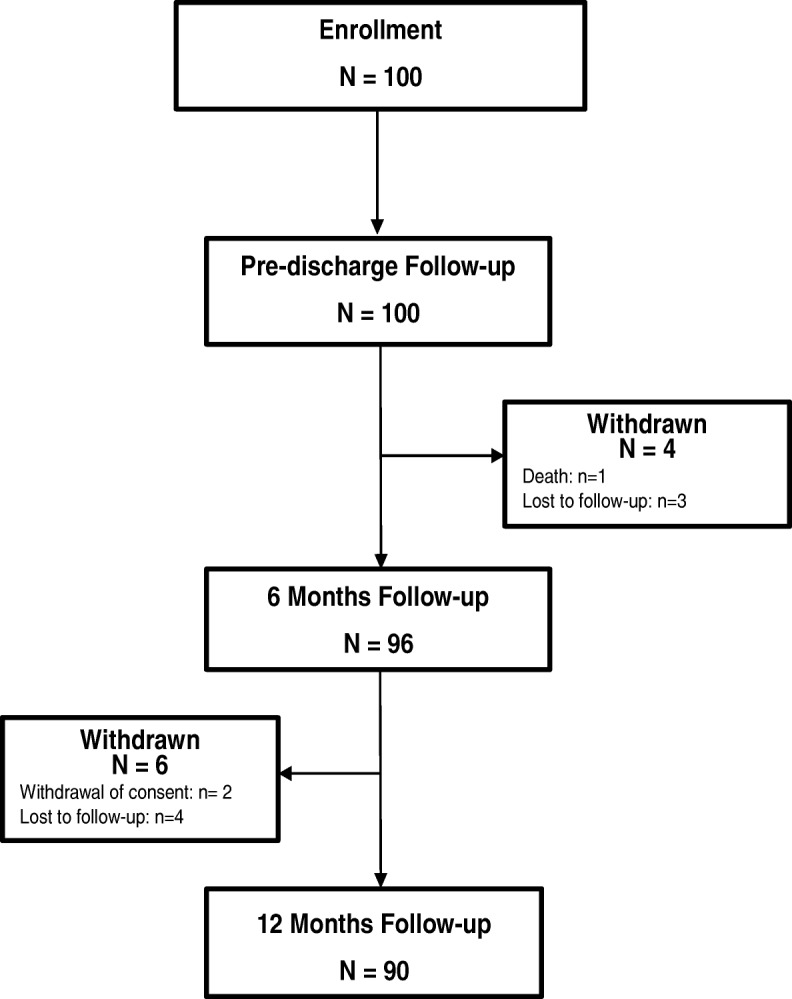
Study Flow Chart

Procedural outcomes are summarized in Table 2. All prostheses were implanted in a supra-annular position, with the majority of surgeons using a median sternotomy surgical approach (77%) and simple interrupted suture technique (51%). Thirty-three patients underwent a concomitant surgical procedure; with 28% requiring coronary artery bypass grafting. Average cardiopulmonary bypass time was 112.1 ± 49.2 min and average aortic cross clamp time was 78.4 ± 33.8 min. Patients were predominantly implanted with a 19 mm (*n* = 36) or 21 mm (*n* = 45) Trifecta valve. For the purposes of subsequent analyses, all patients implanted with a 23 mm (*n* = 13), 25 mm (*n* = 5) and 27 mm valve (*n* = 1) have been grouped into a single cohort. There were no instances of intra-procedural mortality. A total of 90 patients completed the 12-month follow-up visit (90%; Fig. 1: Flow chart). The remaining 10 subjects either, were lost to follow-up (*n* = 7) or formally withdrew consent (*n* = 2) and died (*n* = 1). The percentage of patients receiving anticoagulation therapy at different visits is shown in Table 3. At baseline 81%, pre-discharge 95%, 6 months 91% and 88% at 12 months were receiving anticoagulation therapy.

**Table 2:** Procedural Characteristics

	All (*n* = 100)
Cardiopulmonary bypass time (min)	112.1 ± 49.23 (98)
Aortic cross clamp time (min)	78.35 ± 33.77 (98)
Suture technique, n (%)	
Simple interrupted	51/100 (51%)
Continuous	3/100 (3%)
Everting mattress	7/100 (7%)
Non-everting mattress	22/100 (22%)
Other	18/100 (18%)
Concomitant surgical procedure, n (%)	
Coronary artery bypass graphing	28/100 (28%)
Mitral valve repair	2/100 (2%)
Other	3/100 (3%)
Implanted valve size	
19 mm	36
21 mm	45
23 mm	13
25 mm	5
27 mm	1

*n* Total number of patients, *min* Minutes, *mm* Millimeter

**Table 3:** Anticoagulants regimen

Visits	Anticoagulant regimen *n* (%)
Baseline	81/100 (81%)
Pre-discharge	95/100 (95%)
6 Month	87/96 (91%)
12 Month	79/90 (88%)

*n* = Total number of patients

### Valve hemodynamic performance

Analyzable echocardiographic data was available for 81 patients (90%; echocardiographic images for 9 patients were not as per the Core Laboratory requirements) at 12 months follow-up.

A consistent low mean pressure gradient across the prosthesis was observed, which was maintained till 12 months of follow-up. Baseline, pre-discharge and 12 month’s echocardiographic parameters according to valve size are summarized in Table 4. The average mean pressure gradients were 51.47 ± 18.34, 10.44 ± 4.40 and 10.42 ± 4.77 at baseline, pre-discharge & 12 months respectively. Results for effective orifice area and effective orifice area index are shown in Table 4. The average effective orifice area was 0.75 ± 0.42, 1.59 ± 0.37 and 1.61 ± 0.30 at baseline, pre-discharge & 12 months respectively The average cardiac was 4.46 ± 2.39 at the time of baseline, 4.45 ± 2.03 at pre-discharge and between 4.85 ± 1.69 at 12 months. (Table 4). Performance Index was calculated at baseline and pre-discharge and at 12 month and is depicted in Table 4. The average performance index was 0.22 ± 0.11 at the time of baseline, 0.47 ± 0.11 at pre-discharge and 0.48 ± 0.08 at 12 months. The average indexed EOAI for all the valves is 0.91 ± 0.19 cm^2^/m^2^ which is above the PPM threshold of ≤0.85 cm^2^/m^2^ with a significance *p*-value of < 0.0001. Hence, no prosthetic patient mismatch was observed (Table 4).

**Table 4:** Pre-Implant and Pre-Discharge Echocardiographic Parameters

Parameters	Baseline	Pre-Discharge	12 Months Follow-Up	*p*-value
19 mm				
Mean Pressure Gradient (mmHg)	53.07 ± 20.21	10.39 ± 3.94	10.89 ± 5.76	< 0.0001
EOA (cm^2^)	0.67 ± 0.24	1.53 ± 0.28	1.58 ± 0.14	< 0.0001
EOA Index (cm^2^/m^2^)	0.4 ± 0.14	0.93 ± 0.20	0.95 ± 0.14	< 0.0001
Cardiac Output (l/min)	4.00 ± 1.98	4.42 ± 2.05	5.12 ± 1.64	0.2698
Cardiac Index (l/min/m^2^)	2.42 ± 1.17	2.7 ± 1.35	3.05 ± 1.05	0.4247
Performance Index	0.24 ± 0.09	0.54 ± 0.10	0.56 ± 0.05	< 0.0001
21 mm				
Mean Pressure Gradient (mmHg)	51.26 ± 16.91	11.4 ± 5.00	10.71 ± 4.41	< 0.0001
EOA (cm^2^)	0.75 ± 0.37	1.55 ± 0.35	1.56 ± 0.13	< 0.0001
EOA Index (cm^2^/m^2^)	0.43 ± 0.24	0.87 ± 0.24	0.87 ± 0.13	< 0.0001
Cardiac Output (l/min)	4.71 ± 2.64	4.29 ± 2.23	4.83 ± 1.57	0.1918
Cardiac Index (l/min/m^2^)	2.88 ± 2.06	2.44 ± 1.32	2.67 ± 0.79	0.1067
Performance Index	0.22 ± 0.11	0.45 ± 0.10	0.45 ± 0.04	< 0.0001
23/25/27 mm				
Mean Pressure Gradient (mmHg)	48.95 ± 18.57	8.25 ± 2.76	8.96 ± 3.75	< 0.0001
EOA (cm^2^)	0.9 ± 0.68	1.78 ± 0.52	1.76 ± 0.60	< 0.0001
EOA Index (cm^2^/m^2^)	0.49 ± 0.35	0.99 ± 0.52	0.96 ± 0.32	< 0.0001
Cardiac Output (l/min)	4.74 ± 2.50	4.9 ± 1.43	4.48 ± 2.08	0.806
Cardiac Index (l/min/m^2^)	2.64 ± 1.35	2.73 ± 0.83	2.47 ± 1.13	0.810
Performance Index	0.2 ± 0.13	0.41 ± 0.10	0.41 ± 0.10	< 0.0001
Total (All Valves)				
Mean Pressure Gradient (mmHg)	51.47 ± 18.34	10.44 ± 4.40	10.42 ± 4.77	< 0.0001
EOA (cm^2^)	0.75 ± 0.42	1.59 ± 0.37	1.61 ± 0.30	< 0.0001
EOA Index (cm^2^/m^2^)	0.43 ± 0.24	0.91 ± 0.24	0.91 ± 0.19	< 0.0001
Cardiac Output (l/min)	4.46 ± 2.39	4.45 ± 2.03	4.85 ± 1.69	0.9254
Cardiac Index (l/min/m^2^)	2.67 ± 1.66	2.59 ± 1.25	2.75 ± 0.97	0.6921
Performance Index	0.22 ± 0.11	0.47 ± 0.11	0.48 ± 0.08	< 0.0001

*mmHg* Millimeter of Mercury, *cm*^*2*^ Centimeter^2^, *cm*^*2*^*/m*^*2*^ Centimeter^2^/Meter^2^, *l/min* Liter/Minute, *l/min/m*^*2*^ Liter/Minute/Meter^2^

**Fig. 2:**
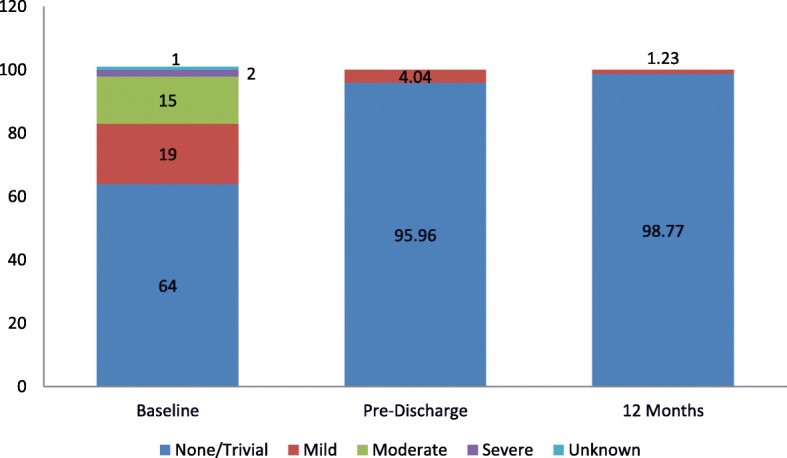
Summary Statistics for incidence/severity of aortic insufficiency Measurements

Aortic regurgitation was observed at baseline in 36 patients (Mild to Severe), at pre-discharge in 4 patients (mild) and at 12 month in 1 patient (mild); none of the patients had moderate or severe aortic regurgitation at pre-discharge and 12 month. (Fig. 2: Summary Statistics for incidence/severity of aortic insufficiency Measurements).

### Clinical events

At 12 months, no incidence of structural valve deterioration, valve thrombosis, or hemolysis was observed. Nine subjects reported 9 separate SAEs events including: hepatic encephalopathy, post pericardiotomy effusion, cerebrovascular accident/intracerebral hemorrhage, complete heart block, metastatic lymph nodes, peripheral vertigo, ventricular arrhythmias, decreased sodium levels and increased total white blood cells and low tract respiratory infection. No SAEs were deemed to be valve related. A single patient death (1%) was reported 37 days post-implant, and was deemed related to a low sodium level (no valvular/para valvular leak was observed in pre-discharge echocardiographic images and had a well- functioning prosthetic aortic valve).

### Change in NYHA functional status

**Fig. 3:**
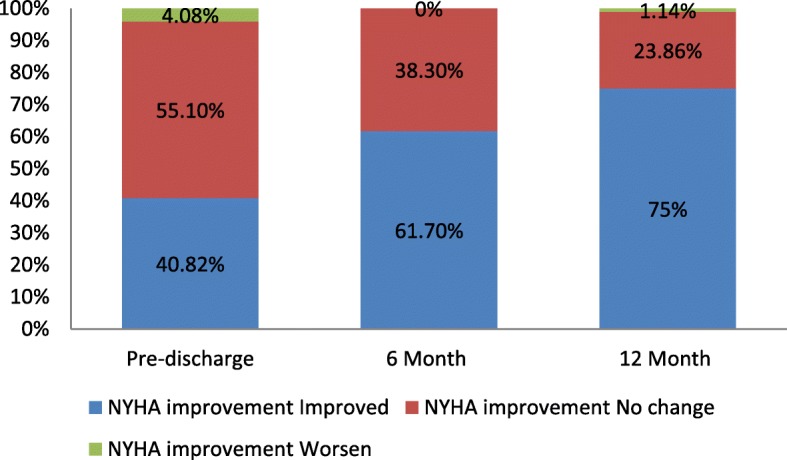
NYHA functional status at pre-discharge, 6 months and 12 months. NYHA: New York Heart Association

In 88 (NYHA data not available for 2 patients) patients with available data at 12 months, NYHA functional status improved by ≥1 class in 75% of patients (66/88) compared to baseline. Only 1 patient reported a worsening in NYHA function class at 12 months compared to baseline (mild AR was observed at 12 months); the remaining 23.9% of patients experienced no change. (Fig. 3: Changes in NYHA functional status at pre-discharge, 6 months and 12 months compared to baseline) summarizes the proportion of patients that improved, did not change, or worsened in NYHA functional status at pre-discharge, 6 months and 12 months compared to baseline.

## Discussion

According to the guidelines of the American Heart Association/American College of Cardiology (AHA/ACC), bioprostheses have been chosen as the most appropriate aortic valve treatment for patients older than 65 years with severe valve stenosis [16]. It is predictable that the number of AVRs with bioprosthesis will become more frequent in future owing to growth of aging population. This is further relevant for developing countries like India where majority of the population cannot afford Transcatheter aortic valve replacement therapy and would rely on surgical approach for treatment of aortic valve disease.

The present prospective, multicenter study evaluated the safety and hemodynamic performance of the Trifecta™ valve across 10 centers in India over a 12 month follow-up duration. The study showed significant portion of patients within smaller age group (mean age of 64 years), 81% had smaller valve size of 19 & 21 mm. The patients with rheumatic heart disease were relatively smaller (14%) owing to study being conducted at big cities across India with relatively better hygiene conditions. The data was in line with previously reported literature [2, 3, 7, 8, 11, 12].

The Trifecta valve was associated with a good safety profile with no incidences of valve thrombosis, clinically significant hemolysis or structural deterioration reported during the 12-month follow-up period. No patient reported any valve-related perioperative complications and overall survival at 12 months was 99% (One death), which is in line with previous reported data [7–10] for patients implanted with the Trifecta valve.

The data from present study establishes acute safety of Trifecta™ Valve in Indian patients.

In previous studies, it has been demonstrated that the external mounting of leaflets in Trifecta™ valve allow for a wider opening, and the expansible stent could limit impedance to flow during high flow conditions as during exercise [17]. The present study demonstrated excellent early hemodynamic performance of the Trifecta™ valve across 12 month follow-up. In the study, the mean pressure gradient for each sized valve was 10.89, 10.71 and 8.96 mmHg for the 19, 21, and (23 + 25) mm valve sizes respectively which compares favourable with previously published data in 1022 patients implanted with a Trifecta valve [18]. Similar results were also reported by Dell’aquila and colleagues [19] who studied 70 patients undergoing SAVR with the Trifecta™ valve. Echocardiographic data at discharge showed that the mean pressure gradient was 14.4, 11.1, and 10.9 mmHg for the 19, 21, and 23 mm valve sizes respectively. The results of a multicenter study by Bavaria and colleagues [7] evaluating 1014 patients undergoing AVR with the Trifecta™ valve were recently reported. In that report, echocardiographic data at discharge and at 12 month showed that mean PG was 9.3/10.7, 7.8/8.1, and 7.3/7.2 mmHg for the 19, 21, and 23 mm valve sizes respectively [7].

In our study, EOA for each size valve was 1.58, 1.56, and 1.76 cm^2^ for the 19, 21, and 23 mm valve sizes, respectively. These data suggest that Trifecta™ valve has a large EOA, similar to previous data (1.41, 1.63, and 1.81 cm^2^ for the 19, 21, and 23 mm valve sizes, respectively) and multicenter study by Bavaria and colleagues (1.58, 1.77, and 1.94 cm^2^ for the 19, 21, and 23 mm valve sizes, respectively) [7].

Currently, the importance of avoiding prosthesis patient mismatch (PPM) (i.e. effective prosthetic valve area, after implantation, which is less than that of a normal human valve) is widely accepted [20–23]. The best variable for defining PPM is ratio of prosthetic EOA to BSA i.e. EOAI< 0.85 cm^2^/m^2^ which is the common cut-off value for all types of prosthetic valves. In our study the average EOAI observed for 19 mm valve size is 0.95 ± 0.14 cm^2^/m^2^, 21 mm valve size is 0.87 ± 0.13 cm^2^/m^2^ and 23/25/27 mm is 0.96 ± 0.32 cm^2^/m^2^. The Trifecta™ valve demonstrates excellent hemodynamic performance on this point and no PPM observed in our study in patients with smaller annulus which is in line with the previous studies’ result.

The study also showed patients had significant improvement with respect to functional class over period of 12 months (75% showed improvement).

With the availability of the Trifecta™ valve, several bioprostheses, options are now available, Carpentier- Edwards Perimount (CEP, Edwards Lifesciences, Irvine, CA, USA); CEP Magna (CEPM, Edwards Lifesciences); Mosaic (Medtronic, Minneapolis, MN, USA); Mosaic Ultra (Mosaic U, Medtronic). However, the performance of these valves in Indian scenario over a long term follow-up study is still warranted.

### Limitations

There are few limitations of the present study including limited number of patients’ enrolled and short follow-up duration without any comparator arm. In the study, fractional shortening was used to evaluate left ventricular function instead of ejection fraction. Left ventricular mass regression was also not assessed.

## Conclusion

This study reports excellent early clinical and hemodynamic performance of the Trifecta™ valve in an Indian patient population. Importantly, no procedural mortality or structural valve deterioration was reported.

### Endnotes

The current study evaluated 12 months follow-up data of   hemodynamic performance of Trifecta™ valve however a long term follow-up study is required in India.

## Appendix

**Table 5:** Subject Enrollment according to Investigational Site (*n* = 10)

Investigational site	Principal Investigator	Subject Enrollment
Star Hospital	Mannam, Gopichand	26
Yashoda Hospital	Gokhale, Alla Gopala	14
Escorts Heart Institute and Research Centre	Mishra, Yugal	13
The Madras Medical Mission	Sethuratnam, Rajan	12
SAL Hospital	Modi, Rajan	11
P D Hinduja Hospital and Medical Research Center	Pandey, Kaushal	10
Max Super Specialty Hospital	Malhotra, Rajneesh	5
Apollo Gleneagles Hospitals	Mukhopadhyay, Sushan	4
Care Institute of Medical Sciences	Shah, Dhiren	4
Fortis Hospital	Mahant, Tek Singh	1
Total		100

## Abbreviations

ACC: American College of Cardiology
AHA: American Heart Association
AR: Aortic Regurgitation
AVR: Aortic valve replacement
BSA: Body surface area
CAD: Coronary Artery Disease
CEP: Carpentier- Edwards Perimount
CEPM: Carpentier- Edwards Perimount Magna
EOA: Effective orifice area
EOAI: Effective orifice area index
IEC: Institutional Ethics Committee
IRB: Institutional Review Board
LVM: Left ventricular mass
NYHA: New York Heart Association
PG: Pressure Gradient
PPM: Prosthesis patient mismatch
RHD: Rheumatic Heart Disease
SVD: Structural Valve Deterioration
TIA: Transient Ischemic Attack
TPG: Trans pulmonary pressure gradient
VARC: Valve Academic Research Consortium
